# Longan flower water extract promotes sleep in mice via the serotonin–melatonin axis

**DOI:** 10.1002/jsfa.70294

**Published:** 2025-11-05

**Authors:** Tzu‐Huan Hung, Chiu‐Ping Cheng, Wei‐Chung Chiou, Tzu‐Lan Hsia, Hui‐Kang Liu, Hsu‐Feng Lu, Yu‐Heng Lai, Shang‐Ting Tsai, Chiung‐Ju Chen, Cheng Huang

**Affiliations:** ^1^ Institute of Plant Biology, College of Life Science National Taiwan University Taipei Taiwan; ^2^ Crop Genetic Resources and Biotechnology Division Taiwan Agricultural Research Institute Taichung Taiwan; ^3^ Department of Biotechnology and Laboratory Science in Medicine National Yang Ming Chiao Tung University Taipei Taiwan; ^4^ Division of Basic Chinese Medicine National Research Institute of Chinese Medicine, Ministry of Health and Welfare Taipei Taiwan; ^5^ PhD Program in Clinical Drug Development of Herbal Medicine, College of Pharmacy Taipei Medical University Taipei Taiwan; ^6^ Department of Medical Laboratory Science and Biotechnology Asia University Wufeng Taiwan; ^7^ Department of Chemistry Chinese Culture University Taipei Taiwan; ^8^ Department of Applied Chemistry National Chiayi University Chiayi Taiwan; ^9^ Department of Pathology and Laboratory Shin Kong Wu Ho‐Su Memorial Hospital Taipei Taiwan

**Keywords:** longan flower water extract, sleep regulation, serotonin, melatonin, polyphenols, LC–MS profiling

## Abstract

**BACKGROUND:**

Longan (*Dimocarpus longan*) flower is an underutilized botanical byproduct rich in polyphenols with reported neuroprotective actions, yet its role in sleep regulation remains unclear. This study aimed to evaluate the sleep‐promoting effects of longan flower water extract (LFWE) and its underlying neurochemical mechanisms *in vivo*. Although longan flower extract has been suggested to enhance melatonin biosynthesis *in vitro*, its functional role in sleep modulation remains unclear.

**RESULTS:**

High‐resolution liquid chromatography–mass spectrometry profiling demonstrated that LFWE is rich in phenolics and flavonoids, with corilagin, gallic acid and epicatechin as dominant constituents, alongside ellagic acid, rutin, procyanidin A2, quercetin and kaempferol. Male C57BL/6 mice received oral LFWE (25 mg kg^−1^ d^−1^) for 7 or 14 days. Sleep behavior was assessed in an isoflurane‐induced sleep paradigm using loss‐of‐righting‐reflex endpoints. LFWE significantly prolonged total sleep duration after 7 and 14 days relative to vehicle, while sleep latency was unchanged, indicating preferential effects on sleep maintenance rather than initiation. To explore mechanisms, neurotransmitters in brain homogenates and melatonin in plasma were quantified by enzyme‐linked immunosorbent assay on day 14. LFWE elevated brain serotonin and dopamine and increased circulating melatonin compared with vehicle.

**CONCLUSION:**

Our findings support a mechanistic model in which LFWE augments the serotonin–melatonin axis and modulates monoaminergic tone to enhance sleep maintenance. The combination of water solubility, high polyphenol content and selective prolongation of sleep duration without sedative‐like latency reduction highlights LFWE as a safe and sustainable phytochemical resource with translational potential in functional food and nutraceutical applications for sleep regulation. © 2025 The Author(s). *Journal of the Science of Food and Agriculture* published by John Wiley & Sons Ltd on behalf of Society of Chemical Industry.

## INTRODUCTION

Sleep is a critical physiological process essential for cognitive function, emotional regulation and systemic homeostasis. Both insufficient sleep duration and compromised sleep quality are increasingly recognized as risk factors for chronic conditions such as cardiovascular disease, metabolic disorders and immune dysfunction.^1^, ^2^ Insomnia, the most prevalent sleep disorder, is defined by difficulty initiating or maintaining sleep and is frequently accompanied by daytime impairment, mood disturbances or comorbid depression.^3^, ^4^, ^5^ A recent global systematic review estimated that approximately 16.2% of adults suffer from insomnia, with 7.9% affected by severe forms of the disorder.^6^ This high global burden underscores insomnia as a public health concern with significant medical, psychological and socioeconomic implications.

Current pharmacological treatments for insomnia – such as benzodiazepines, non‐benzodiazepine hypnotics (Z‐drugs) and melatonin receptor agonists – primarily act on GABAergic or circadian pathways.^7^, ^8^ Although these kinds of drugs are effective, long‐term use of benzodiazepines is associated with substantial risks of dependency and residual sedation.^9^, ^10^ Z‐drugs are generally considered safer but still carry concerns regarding tolerance and next‐day drowsiness.^10^ Melatonin receptor agonists, though exhibiting lower dependency potential, may cause mild adverse effects such as daytime somnolence or dizziness in some individuals.^11^ These safety considerations have driven increasing interest in plant‐derived bioactive compounds that may modulate sleep through safer, multi‐target mechanisms.^12^, ^13^

Several phytochemicals, particularly phenolic acids and flavonoids, have been reported to show sedative or sleep‐regulating effects through diverse mechanisms, including modulation of monoaminergic neurotransmission, enhancement of melatonin biosynthesis, attenuation of oxidative stress and interaction with GABAergic pathways.^14^, ^15^, ^16^, ^17^ These compounds are widely distributed in edible plants and traditional medicinal herbs, many of which have long been used in the form of teas, decoctions or functional beverages to promote sleep and relaxation.^12^, ^18^ In recent years, there has been increasing interest in repurposing underutilized botanical materials – especially agricultural byproducts rich in polyphenols – as renewable sources of bioactive agents for sleep support,^18^, ^19^, ^20^ reinforcing the potential of phytochemical‐rich plant extracts in nutraceutical development.

Longan (*Dimocarpus longan* Lour.), a member of the Sapindaceae family, is widely cultivated in tropical and subtropical regions of Southeast Asia, such as Taiwan, Thailand, Vietnam and southern China.^21^, ^22^ Longan pulp is a common traditional Chinese medicine, known for its calming and tonic effects.^22^ Modern research has identified a range of biological activities of longan pulp, including antioxidant, immunomodulatory and neuroprotective properties.^23^, ^24^, ^25^ In recent years, the bioactive potential of longan byproducts has attracted attention.^22^, ^26^ Longan flowers – often discarded during fruit production – have been identified as a rich source of flavonoids and phenolic acids.^27^, ^28^ Early studies revealed that longan flower water extract (LFWE) exhibits potent antioxidant and anti‐inflammatory effects, including suppressing nitric oxide and prostaglandin E₂ production in activated macrophages and inhibiting lipid peroxidation in neuronal tissues.^27^, ^29^ A recent conference abstract reported that oral administration of longan flower extract in adults with mild insomnia enhanced melatonin and serotonin levels, and improved sleep quality according to standardized questionnaires.^30^ *In vitro* cell experiments further demonstrated that the extract upregulated melatonin biosynthetic genes and reduced oxidative and inflammatory markers.^31^ These findings suggest that longan flowers may be relevant to sleep regulation. Despite these interesting findings from *in vitro* and preliminary human studies, the functional relevance of longan flowers in sleep modulation remains underexplored, particularly in validated *in vivo* models.

The study reported here aimed to evaluate the sleep‐promoting potential of LFWE using an isoflurane‐induced sleep model in C57BL/6 mice – a validated behavioral paradigm for assessing sleep latency and duration. We assessed its effects on sleep behavior, neurotransmitter levels and melatonin secretion. In parallel, high‐resolution liquid chromatography (LC)–mass spectrometry (MS) profiling was conducted to characterize its major phytochemical constituents. The findings offer mechanistic insights and preclinical evidence supporting the potential application of LFWE as a functional food or nutraceutical for sleep modulation.

## MATERIALS AND METHODS

### Preparation of LFWE

Fresh longan flowers were purchased directly from local farmers in Chiayi County, Taiwan (GPS: 23.500930° N, 120.532471° E). The plant material was authenticated by Dr. Jer‐Way Chang at the Chiayi Agricultural Experiment Branch, Taiwan Agricultural Research Institute (TARI). A voucher specimen (batch no. TARI‐23L04) has been deposited at TARI for future reference. The harvested flowers were sun‐dried for three days. Moisture content was measured using a moisture analyzer (MX‐50, AND, Japan) to ensure it was below 10%. Dried flowers were extracted with ultrapure water at a 1:2 (w/w) ratio using ultrasonic treatment at 60 °C for 1 h with a sonicator (ES‐600N, TST, Taiwan; frequency 40 kHz, power 600 W). The extract was subsequently filtered through Whatman No. 1 filter paper to remove particulates and then concentrated under reduced pressure using a rotary evaporator. The concentrated extract was lyophilized using a freeze dryer (FD‐25B3P8, HCS, Taiwan) to yield a fine powder. This lyophilized extract powder was stored at −20 °C in airtight containers until further analysis. For all experiments, the lyophilized extract was freshly reconstituted in distilled water to the desired concentrations and was completely soluble without visible precipitation. LFWE refers to the reconstituted aqueous extract derived from the lyophilized powder.

### Determination of total phenolic content, total flavonoid content and antioxidant capacity

The total phenolic content was determined using the Folin–Ciocalteu method.^32^ Briefly, LFWE was mixed in a 1:1 ratio with 0.5 N Folin–Ciocalteu reagent (Merck, Germany, catalog number 109001). After a pre‐incubation at room temperature, an equal volume of 20% sodium carbonate solution (Honeywell, Germany, catalog number 31432) was added. The resulting mixture was analyzed for absorbance at 750 nm using a microplate reader (SpectraMax 250, Molecular Devices, USA). A calibration curve was constructed using gallic acid (Thermo Fisher Scientific, USA, catalog number 410862500) as the standard, at concentrations ranging from 0 to 100 μg mL^−1^. Total phenolic content was expressed as milligrams of gallic acid equivalent (GAE) per gram of extract (mg GAE g^−1^ extract).

The total flavonoid content was analyzed following a method adapted from Jing *et al*.,^33^ with minor modifications. Briefly, LFWE was mixed in a 4:1 ratio with 2% sodium nitrite solution (Merck, Germany, catalog number 106549). Subsequently, 1/5 volume of 4% aluminium chloride solution (JT Baker, USA, catalog number 0528‐01) was added. Afterward, 1/3 volume of 4% sodium hydroxide solution was introduced to the mixture. The absorbance at 510 nm was measured using a microplate reader (SpectraMax 250, Molecular Devices, USA). A calibration curve was constructed using rutin (Cayman, USA, catalog number 19868) as the standard, at concentrations ranging from 0 to 500 μg mL^−1^. Total flavonoid content was expressed as milligrams of rutin equivalent (RUE) per gram of extract (mg RUE g^−1^ extract).

Antioxidant activity was evaluated using the 2,2′‐azinobis(3‐ethylbenzothiazoline‐6‐sulfonic acid) (ABTS) radical scavenging assay, with the diluted ABTS^•+^ radical solution prepared as described previously.^34^ For the antioxidant activity analysis of LFWE, the diluted ABTS^•+^ radical solution was mixed in a 9:1 ratio with LFWE or water (for the control group), and the absorbance was immediately recorded at 734 nm using a microplate reader. A calibration curve was constructed using Trolox (Cayman, USA, catalog number 10011659) as the standard, at concentrations ranging from 0 to 60 μg mL^−1^. Antioxidant activity was expressed as Trolox equivalent antioxidant capacity (TEAC) per gram of extract (mg TEAC g^−1^ extract).

### High‐resolution LC–MS analysis

Chromatographic separation was performed using a Thermo Ultimate 3000 UHPLC System (Thermo Fisher Scientific, USA) equipped with an ACQUITY UPLC HSS T3 column (2.1 × 100 mm, 1.8 μm; Waters, USA). The elution was carried out using a gradient program as follows: 0–2 min, isocratic 95% A (2% acetonitrile–formic acid, 99.9/0.1 (v/v)), 5% B (acetonitrile–formic acid, 99.9/0.1 (v/v)); 2–15 min, linear from 5% to 20% B; 15 to 22 min, linear from 20% to 80% B; 22 to 27 min, linear from 80% to 99.9% B; 27 to 28.5 min, isocratic 99.9% B; 28.5 to 28.6 min, linear from 99.9% to 5% B; finally, 28.5 to 30 min, isocratic 5% B. The flow rate was maintained at 0.4 mL min^−1^, and the column temperature was held at 40 °C.

MS analysis was conducted using an Orbitrap Fusion Tribrid mass spectrometer (Thermo Fisher Scientific, USA) equipped with an electrospray ionization source operating in negative ion mode. The instrument parameters were set as follows: sheath gas flow rate, 50 arbitrary units; auxiliary gas flow rate, 10 arb; sweep gas flow rate, 3 arb; spray voltage, 2.5 kV; ion transfer tube temperature, 325 °C; vaporizer temperature, 350 °C. Full‐scan mass spectra were acquired in the range *m*/*z* 100–1200. MS/MS spectra were acquired using higher‐energy collisional dissociation with stepped collision energy of 35 ± 15%.

A total of eight standard compounds – corilagin, gallic acid, epicatechin, ellagic acid, procyanidin A2, kaempferol (from Sigma‐Aldrich, USA), along with rutin and quercetin (from ChromaDex, USA) – were used for identification and quantification. These analytical‐grade standards (purity ≥ 98%) were first dissolved in a small volume of dimethyl sulfoxide and combined into a mixed stock solution. Sequential 10‐fold dilutions of the mixed stock solution were then prepared using methanol to generate calibration curves. Quantification of target phytochemicals in LFWE was performed using external standard calibration, and linear regression models for each compound achieved coefficients of determination (*R* ^2^) greater than 0.99 across the working concentration ranges.

### Animals and sleep‐induction protocol

The sleep‐induction protocol was adapted from Murai *et al*.^35^ Briefly, six‐week‐old male C57BL/6 mice were obtained from the National Laboratory Animal Center (Taipei, Taiwan) and housed three per cage under standard conditions with a 12 h light/dark cycle at a controlled temperature of 24 ± 2 °C. Food and water were provided *ad libitum* throughout the experiment. All animal procedures were performed in compliance with the guidelines of the Animal Center of National Yang Ming Chiao Tung University (IACUC approval no. 1121005, 2023), the Guide for the Care and Use of Laboratory Animals (NIH Publication No. 85‐23, revised 1996) and the Animal Welfare Act of Taiwan.

**Figure 1:**
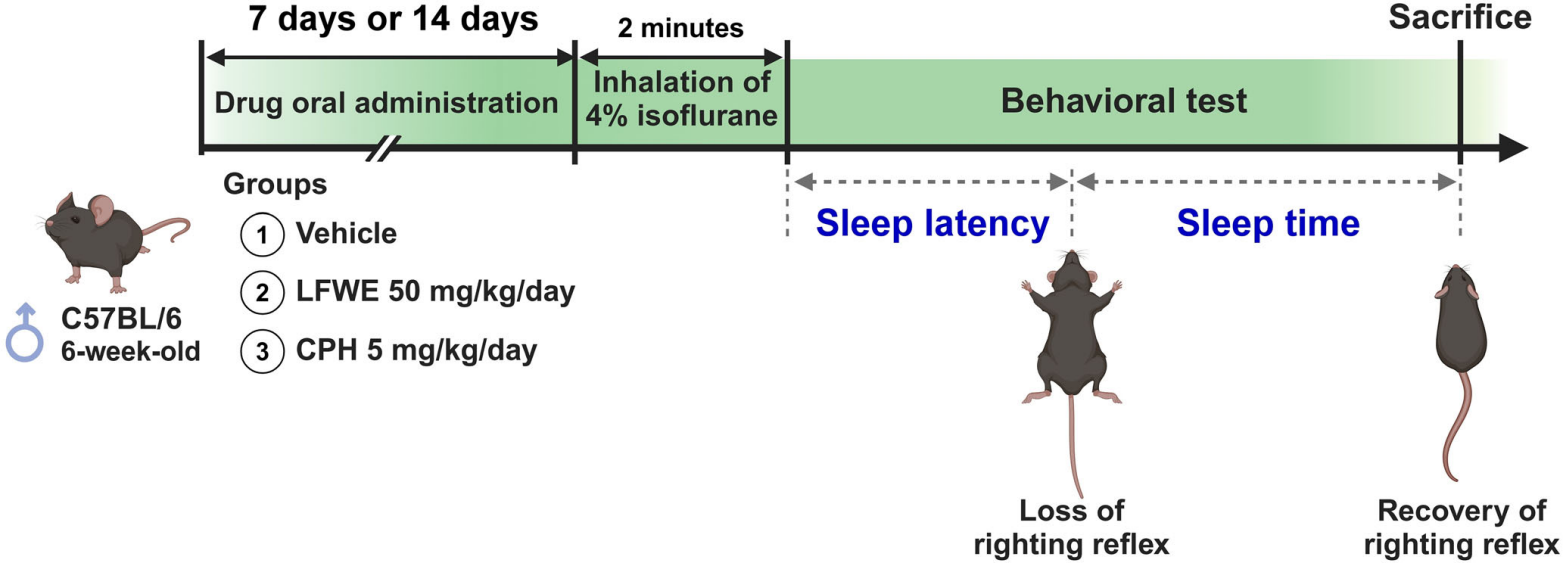
Experimental design for investigating the effects of LFWE on sleep quality in mice. (A) Acute effects of LFWE on sleep facilitation. (B) Chronic effects of LFWE on sleep facilitation. Mice were exposed to 4% isoflurane in airflow for 2 min to induce sleep behavior, either 1 h (A) or 7–14 days (B) after LFWE administration via gastric gavage. Sleep latency and sleep time were assessed based on the righting reflex behavior.

The overall experimental design, including acute and chronic treatment schedules, is illustrated in Fig. 1. Mice were randomly assigned into three experimental groups (*n* = 6 per group): a vehicle control group, a positive control group receiving cyproheptadine (CPH; 5 mg kg^−1^) and an LFWE‐treated group (25 mg kg^−1^). Treatments were administered once daily by oral gavage for either 7 days (acute) or 14 days (chronic) prior to sleep assessment.

To induce sleep, anesthesia was achieved by placing each mouse in a chamber filled with 4% isoflurane in air for 2 min, using a vaporizer (model SN‐487‐0T; Shinano Manufacturing Co. Ltd, Tokyo, Japan). Immediately after removal from the chamber, mice were positioned supine to assess sleep‐related behavioral responses based on the righting reflex. Loss of righting reflex (LORR) was defined as the inability to maintain an upright posture, while recovery was marked by the first successful attempt to regain this posture.^36^ Sleep latency was defined as the interval from removal from the anesthesia chamber to the onset of LORR, and sleep duration was the time from LORR onset to recovery.

Following the assessment, animals were returned to their home cages and monitored until full recovery. Data were recorded and expressed as mean ± standard error of the mean (SEM). At the conclusion of the experiment, mice were deeply anesthetized with an intramuscular injection of ketamine (100 mg kg^−1^ body weight) and xylazine (5 mg kg^−1^ body weight), followed by euthanasia via cardiac puncture and cervical dislocation. Blood and brain tissues were immediately collected for subsequent biochemical analyses.

### Enzyme‐linked immunosorbent assay (ELISA) for brain tissue and plasma

To evaluate the effects of long‐term treatment, all ELISA analyses were performed using samples collected on day 14, following the final administration of LFWE or control treatments. Immediately after euthanasia, whole blood was obtained via cardiac puncture and centrifuged at 2000 × *g* for 15 min at 4 °C to separate plasma. Brains were rapidly harvested and homogenized in cold phosphate‐buffered saline (pH 7.4) containing protease inhibitors (Sigma‐Aldrich, no. 11697498001), using a handheld tissue grinder. The homogenates were centrifuged at 12 000 × *g* for 10 min at 4 °C, and the resulting supernatants were collected for neurotransmitter quantification.

Dopamine and serotonin concentrations were measured from brain tissue homogenates, while melatonin levels were determined from plasma samples. Quantification was performed using commercial ELISA kits, following the manufacturers' protocols: dopamine (E‐EL‐0046‐96T, Elabscience, USA), serotonin (ab133053‐96T, Abcam, UK) and melatonin (ENZ‐KIT150‐0001‐96T, Enzo Life Sciences, USA). All measurements were performed in duplicate. Absorbance was measured using a microplate reader (SpectraMax 250, Molecular Devices, USA), and standard curves were used to calculate concentrations. Neurochemical levels were normalized to total protein content and expressed as pg μg^−1^ protein.

### Statistical analysis

All statistical analyses and graphical visualizations were performed using R version 4.5.0 (R Core Team, Vienna, Austria). Data were assessed for normality and homogeneity of variance prior to analysis. Comparisons among groups were conducted using one‐way analysis of variance (ANOVA), followed by Dunnett's *post hoc* test to determine differences between each treatment group and the vehicle control. Results are expressed as mean ± SEM. A value of *P* < 0.05 was considered statistically significant.

## RESULTS

### Total phenolic and flavonoid contents and antioxidant capacity of LFWE

The phytochemical composition and antioxidant capacity of LFWE were quantitatively assessed. The total phenolic content was 352.7 ± 29.1 mg GAE g^−1^ extract, while the total flavonoid content reached 472.0 ± 60.6 mg RUE g^−1^ extract. In the ABTS radical‐scavenging assay, LFWE exhibited a TEAC of 703.9 ± 56.5 mg TEAC g^−1^ extract (Table 1). These results indicate that LFWE is rich in phenolic and flavonoid compounds and possesses considerable antioxidant potential, which may contribute to its subsequent biological activities.

**Table 1:** Total phenolic content, total flavonoid content and antioxidant activity data of LFWE

Sample	Total phenolic content (mg GAE g^−1^ extract)	Total flavonoid content (mg RUE g^−1^ extract)	Antioxidant activity (mg TEAC g^−1^ extract)
LFWE	352.7 ± 29.1	472.0 ± 60.6	703.9 ± 56.5

GAE, gallic acid equivalent; RUE, rutin equivalent; TEAC, Trolox equivalent antioxidant capacity.

### Characterization of LFWE

**Figure 2:**
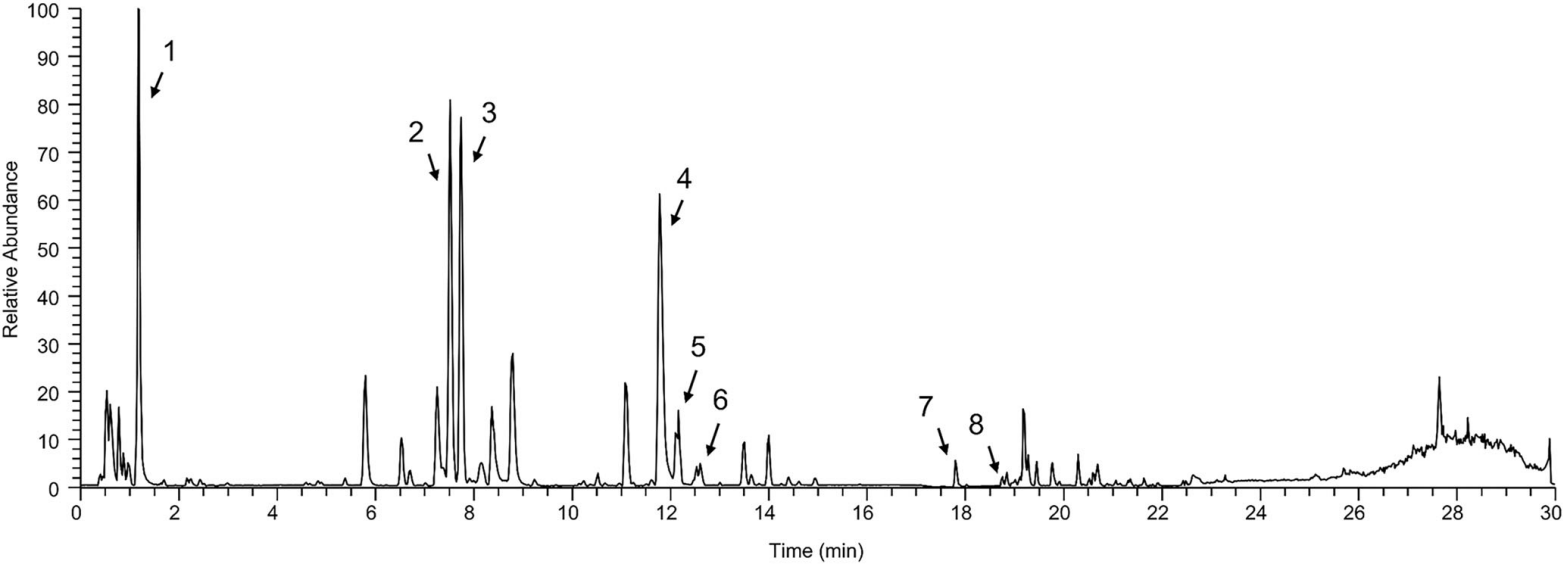
Representative base peak chromatogram of LFWE obtained by LC–MS. Eight peaks corresponding to identified compounds are labeled. Detailed information on retention time, molecular formula and concentration is provided in Table 2.

The chemical composition of LFWE was characterized using LC–MS. The base peak chromatogram of LFWE (Fig. 2) displayed eight major peaks, which were identified based on their retention times, molecular formulas, deprotonated molecular ion signals ([M − H]^−^) and comparison with authenticated standards. As summarized in Table 2, the identified compounds include gallic acid, epicatechin, corilagin, ellagic acid, rutin, procyanidin A2, quercetin and kaempferol. These constituents belong to three major phytochemical classes: phenolic acids (gallic acid), flavonoids (epicatechin, rutin, quercetin, kaempferol) and tannins (corilagin, ellagic acid, procyanidin A2).

**Table 2:** LC–MS‐based identification and quantification of phytochemicals in LFWE. Amounts are expressed as mean ± standard deviation, *n* = 3 (technical replicates)

Peak	Compound	Class of compounds	Retention time (min)	Formula	[M − H]^−^ (*m*/*z*)	Amount (mg g^−1^)
1	Gallic acid	Phenolic acids	1.155	C₇H₆O₅	169.01	33.74 ± 8.52
2	Epicatechin	Flavonoids	7.325	C₁₅H₁₄O₆	289.07	30.25 ± 1.66
3	Corilagin	Tannins	7.512	C₂₇H₂₂O₁₈	633.07	54.61 ± 2.74
4	Ellagic acid	Tannins	11.414	C₁₄H₆O₈	301.00	22.98 ± 0.61
5	Rutin	Flavonoids	11.786	C₂₇H₃₀O₁₆	609.15	4.74 ± 0.24
6	Procyanidin A2	Tannins	12.221	C₃₀H₂₆O₁₂	575.11	3.53 ± 0.04
7	Quercetin	Flavonoids	17.353	C₁₅H₁₀O₇	301.04	0.22 ± 0.02
8	Kaempferol	Flavonoids	18.286	C₁₅H₁₀O₆	285.04	0.08 ± 0.00

Among the identified constituents, corilagin was the most abundant (54.61 ± 2.74 mg g^−1^), followed by gallic acid (33.74 ± 8.52 mg g^−1^) and epicatechin (30.25 ± 1.66 mg g^−1^). Ellagic acid was also present at a moderate level (22.98 ± 0.61 mg g^−1^), while rutin (4.74 ± 0.24 mg g^−1^), procyanidin A2 (3.53 ± 0.04 mg g^−1^), quercetin (0.22 ± 0.02 mg g^−1^) and kaempferol (0.08 ± 0.00 mg g^−1^) were detected in lower concentrations (Table 2).

### Sleep duration

**Figure 3:**
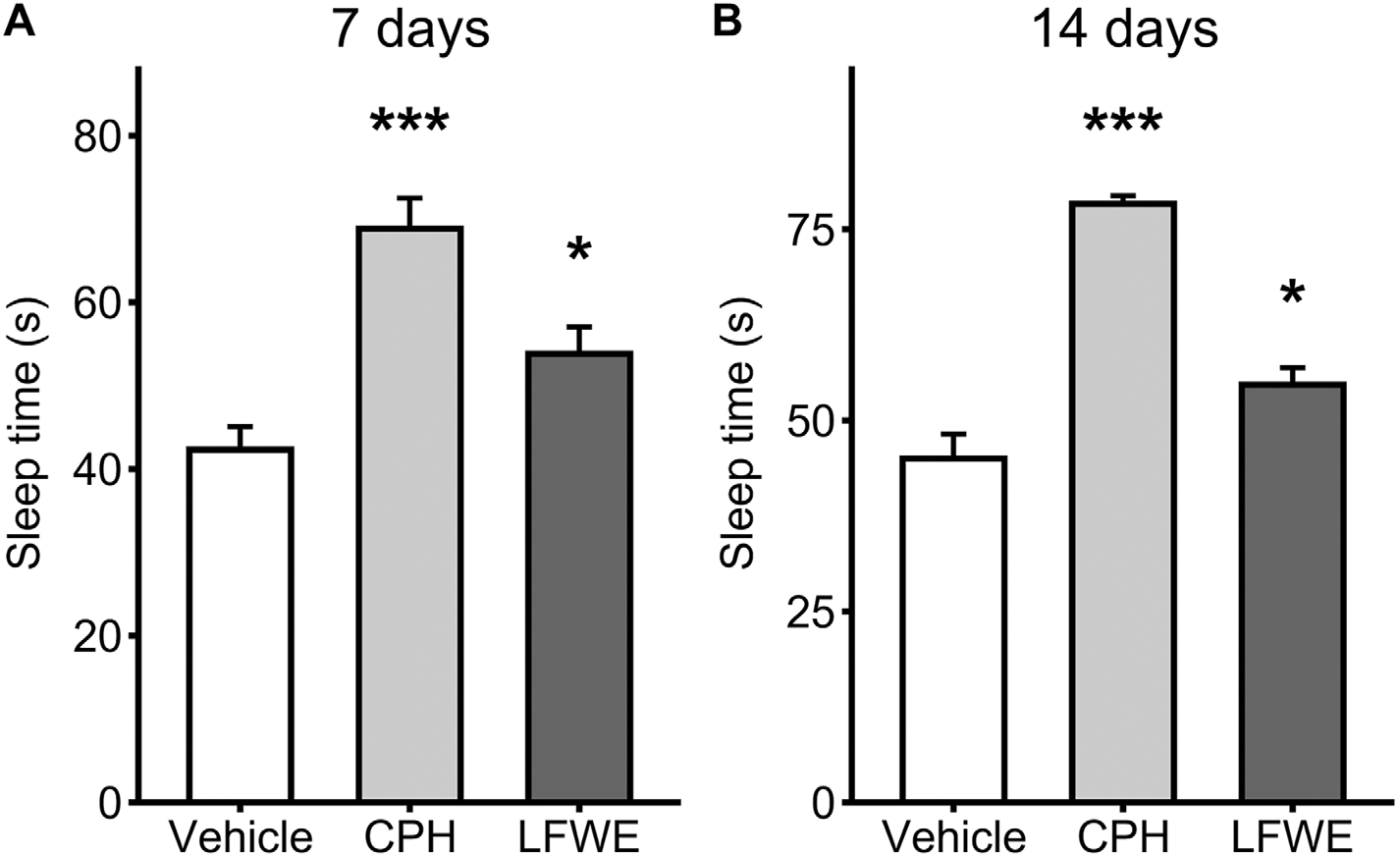
Effects of 7‐day and 14‐day LFWE administration on total sleep duration. Total sleep duration was measured after 7 days (A) and 14 days (B) of treatment with vehicle, LFWE or CPH (positive control). Data are presented as mean ± SEM (*n* = 6 per group). Statistical significance was determined using one‐way ANOVA followed by Dunnett's multiple comparisons test, comparing each treatment group to the vehicle control. Significance levels are indicated as *P < 0.05, **P < 0.01 and ***P < 0.001.

To evaluate the sleep‐promoting potential of LFWE, sleep duration was measured following 7 and 14 days of oral administration. At day 7, both LFWE (53.8 ± 3.6 s, *P* = 0.022) and the positive control CPH (72.2 ± 3.3 s, *P* < 0.001) significantly increased sleep duration compared to the vehicle group (42.3 ± 3.6 s) by Dunnett's multiple comparisons test (Fig. 3(A)). By day 14, sleep duration remained significantly elevated in the LFWE group (54.7 ± 2.1 s, *P* = 0.003) and was further enhanced in the CPH group (78.3 ± 1.9 s, *P* < 0.001), relative to the vehicle control (45.0 ± 2.7 s) (Fig. 3(B)). These results suggest that LFWE exhibits a sustained effect on sleep maintenance, with measurable efficacy evident after short‐term treatment and persisting with continued administration.

### Sleep latency

**Figure 4:**
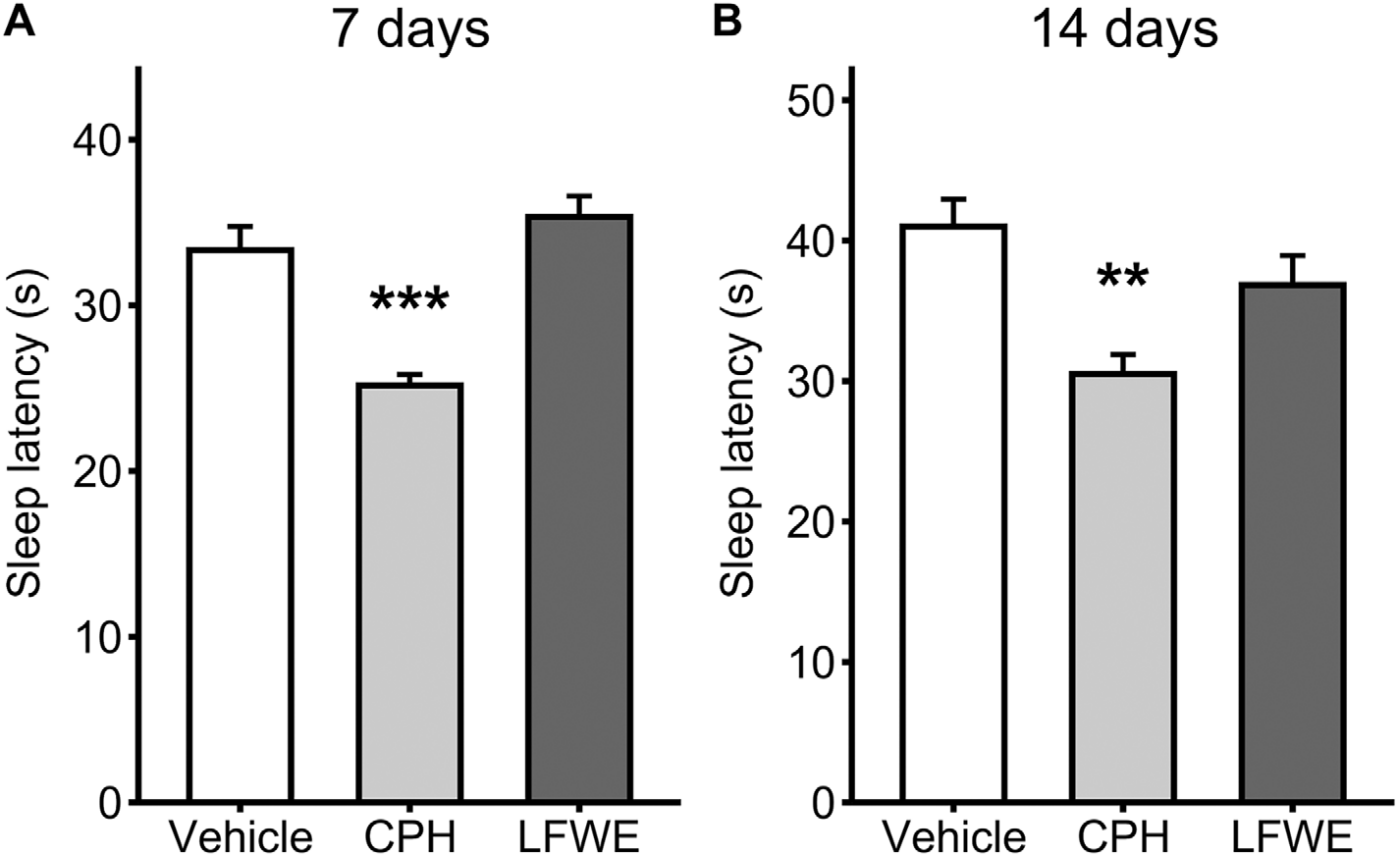
Effects of 7‐day and 14‐day LFWE administration on sleep latency. Sleep latency was measured after 7 days (A) and 14 days (B) of treatment with vehicle, LFWE or CPH (positive control). Data are presented as mean ± SEM (*n* = 6 per group). Statistical significance was determined using one‐way ANOVA followed by Dunnett's multiple comparisons test, comparing each treatment group to the vehicle control. No significant differences were observed for LFWE. Significance levels are indicated as *P < 0.05, **P < 0.01 and ***P < 0.001.

To evaluate the effect of LFWE on sleep onset, sleep latency was measured after 7 and 14 days of administration. At day 7, the positive control CPH significantly reduced sleep latency (25.2 ± 0.7 min, *P* < 0.001) compared to the vehicle group (33.3 ± 1.4 min), while no significant difference was observed in the LFWE group (35.3 ± 1.3 min) (Fig. 4(A)). Similarly, on day 14, CPH continued to significantly shorten sleep latency (30.5 ± 1.4 min, *P* = 0.002) *versus* vehicle (41.0 ± 1.9 min), whereas LFWE again showed no significant effect (36.8 ± 2.1 min) (Fig. 4(B)).

### Effect of LFWE on sleep‐associated endocrines and neurotransmitters in mice

**Figure 5:**
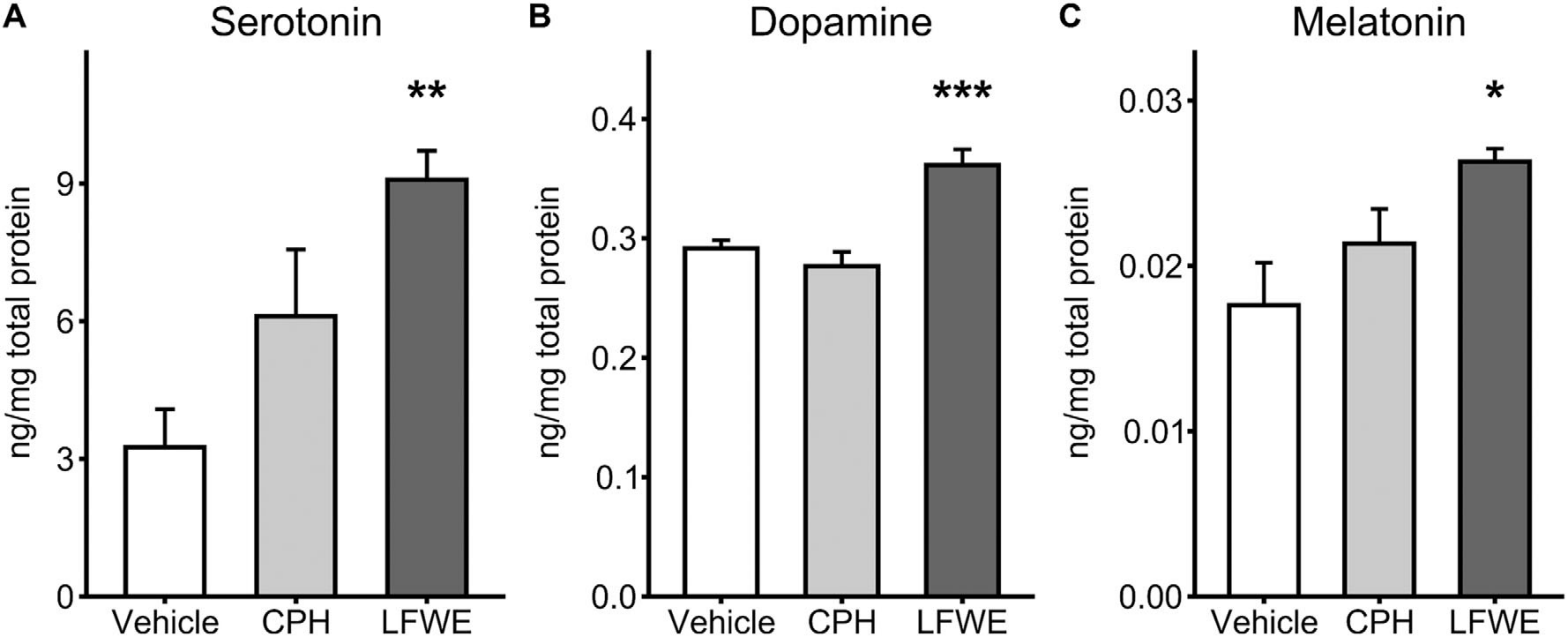
Changes in neurotransmitter and endocrine levels in brain tissue and plasma after 14 days of LFWE administration in sleep‐induced mice. Mouse brain tissue was collected to measure (A) dopamine and (B) serotonin levels, while (C) plasma melatonin concentrations were also determined. Data are presented as mean ± SEM (*n* = 6 per group). Statistical significance was assessed by one‐way ANOVA followed by Dunnett's multiple comparisons test comparing treatment groups to the vehicle control. Statistical significance is indicated as *P < 0.05, **P < 0.01 and ***P < 0.001.

Following 14 days of continuous oral administration, LFWE significantly altered several neurochemical markers associated with sleep regulation. ELISA quantification showed that brain serotonin levels were markedly elevated in the LFWE group (9.08 ± 0.63 ng mg^−1^ total protein, *P* = 0.002) compared to the vehicle group (3.26 ± 0.83 ng mg^−1^), indicating a substantial upregulation of serotonergic tone (Fig. 5(A)). In contrast, serotonin levels in the CPH group (6.11 ± 1.45 ng mg^−1^) did not significantly differ from vehicle.

Dopamine levels were also significantly higher in the LFWE group (0.361 ± 0.013 ng mg^−1^ total protein, *P* < 0.001) than in vehicle‐treated mice (0.292 ± 0.007 ng mg^−1^), suggesting involvement in arousal–sleep balance or reward‐related modulation (Fig. 5(B)). No significant change was observed in the CPH group (0.277 ± 0.012 ng mg^−1^).

Regarding the endocrine marker melatonin, LFWE treatment resulted in a significant increase in circulating plasma levels (0.026 ± 0.001 ng mg^−1^ total protein, *P* = 0.013) relative to the vehicle group (0.018 ± 0.003 ng mg^−1^), implying potential support for circadian rhythm regulation (Fig. 5(C)). CPH did not induce a significant change in melatonin levels (0.021 ± 0.002 ng mg^−1^).

## DISCUSSION

Consistent with previous research,^27^, ^28^ our study indicates that LFWE is a natural plant‐derived extract rich in polyphenols and flavonoids, and exhibits strong antioxidant properties (Table 1). Preliminary *in vitro* and exploratory human studies have suggested the potential sleep‐enhancing properties of longan flower. However, robust *in vivo* evidence, particularly from animal models, to support these claims remains limited. In this study, we demonstrated that both 7‐day and 14‐day administration of LFWE significantly prolonged sleep duration in mice. While no significant differences in sleep latency were observed in this study, some agents, such as selective GABAA agonists or certain plant‐based extracts, show greater efficacy in extending sleep duration than in reducing sleep latency.^37^, ^38^ This is likely due to their specific mechanisms of action, which may not strongly impact the latency phase but can extend total sleep time by stabilizing sleep architecture or enhancing recovery‐related processes.^38^ Such effects could be linked to the indirect modulation of serotonergic signaling, antioxidant defense or melatonergic rhythm regulation, rather than direct sedation. Taken together, the observed enhancement in sleep duration with LFWE highlights its potential to improve overall sleep quality with prolonged use. To our knowledge, this is the first *in vivo* animal study providing preliminary evidence supporting the hypnotic potential of LFWE.

Furthermore, our findings offer novel insights into the possible mechanisms of action of LFWE, particularly through modulation of neurotransmitter signaling pathways. Modulation of the serotonin–melatonin axis may represent a key mechanism underlying the sleep‐enhancing activity of LFWE. Neurochemical analysis showed that LFWE significantly elevated brain levels of serotonin and dopamine, neurotransmitters involved in mood regulation, arousal and circadian control. In particular, serotonin not only regulates central nervous activity but also serves as a biochemical precursor for melatonin synthesis in the pineal gland.^39^, ^40^ The observed increase in serotonin reflects enhanced precursor availability for melatonin synthesis. The elevated circulating melatonin levels in LFWE‐treated mice further support the possible upregulation of the serotonergic‐to‐melatonergic pathway (Fig. 5). Given melatonin's pivotal role in circadian entrainment and sleep–wake transitions, particularly under stress or photoperiod disruption,^41^ LFWE's dual modulation of serotonergic and melatonergic pathways may underlie its observed improvements in sleep quality. Additionally, dopamine elevation may synergistically contribute to LFWE's sleep‐promoting profile by modulating wakefulness and motivational states.^42^

Many polyphenols and flavonoids have been reported to participate in neuromodulatory processes relevant to sleep regulation.^16^, ^43^, ^44^ LC–MS analysis revealed that LFWE is rich in polyphenols and flavonoids, including corilagin, gallic acid, epicatechin, procyanidin A2, rutin, quercetin, ellagic acid and kaempferol (Table 2). Among these, epicatechin and procyanidin A2 are considered as major antioxidative compounds identified in LFWE.^45^ Epicatechin has been reported to enhance monoaminergic neurotransmission and upregulate hippocampal brain‐derived neurotrophic factor (BDNF) expression, thereby influencing synaptic plasticity and circadian rhythm alignment.^46^ The co‐administration of epicatechin and quercetin exhibits synergistic neuroprotective effects.^47^ Corilagin, an abundant hydrolysable tannin in LFWE, is well documented for its anti‐inflammatory and neuroprotective properties.^48^, ^49^ Other constituents such as gallic acid and rutin have demonstrated modulatory effects on central serotonergic pathways and GABAergic signaling, respectively, both of which are critical in sleep regulation.^50^, ^51^ Additionally, ellagic acid and kaempferol contribute antioxidative and anti‐inflammatory activities.^52^, ^53^ Although individual compounds have documented bioactivities, the precise contribution of each component within LFWE remains to be elucidated. The multifactorial mechanism likely involves coordinated modulation of monoaminergic pathways, BDNF expression and oxidative stress reduction – factors closely linked to sleep architecture and quality. Further studies are needed to dissect potential synergistic or additive effects among these phytochemicals, which may collectively underlie LFWE's sleep‐promoting and neuroregulatory functions. On the other hand, our study evaluated LFWE as a whole extract. While several identified constituents, such as gallic acid, epicatechin and rutin, have known neuroactive or antioxidant properties, their individual contributions to sleep modulation remain to be experimentally validated. Investigating the effects of individual compounds or specific fractions represents an important direction for future studies to elucidate causal mechanisms linking specific constituents to observed sleep‐promoting effects.

Isoflurane‐induced sleep differs fundamentally from physiological sleep in terms of cortical activity and neurochemical regulation. Isoflurane primarily acts as a positive allosteric modulator of GABA_A and glycine receptors, inducing hypnosis and immobility through generalized neuronal suppression, rather than the cyclic transitions characteristic of non‐REM and REM stages observed in natural sleep. Thus, while both anesthesia and sleep share key behavioral characteristics such as loss of consciousness and muscle relaxation, anesthesia represents a pharmacologically induced and non‐physiological state. However, the isoflurane‐induced LORR model remains a widely accepted pharmacological tool for evaluating the sleep‐promoting or sedative potential of test compounds. It offers several advantages over barbiturate‐based models, including precise control of anesthetic depth, rapid recovery and minimal interindividual variability. Previous studies have shown that agents acting on GABAergic, benzodiazepine or glycinergic pathways modulate isoflurane anesthesia duration in a manner consistent with their known sedative potency, supporting the model's pharmacological validity for preliminary screening.

In our study, each mouse was exposed to 4% isoflurane for only 2 min during sleep induction. This brief exposure period was chosen to minimize anesthetic stress and avoid prolonged suppression of neuronal activity. All animals recovered spontaneously within a few minutes after removal from the chamber, and no abnormal behavior, mobility or grooming deficits were observed during post‐exposure monitoring. Moreover, sleep assessment was performed only once per animal to eliminate possible cumulative effects of repeated anesthesia. Therefore, the influence of isoflurane on subsequent behavioral or neurochemical parameters is considered minimal under the current experimental conditions. It is worth noting that only male mice were used in this study to minimize hormonal variability associated with the estrous cycle, which can influence sleep architecture and neurochemical responses. However, given the potential for sex‐dependent differences in sleep regulation and behavioral outcomes, future studies including both male and female subjects will be essential to validate these findings and ensure broader translational relevance.

Compared to conventional sedative agents, LFWE may offer a safer, more sustainable alternative for improving sleep maintenance. LFWE demonstrated a gradual but consistent increase in sleep duration without inducing excessive sedation or notable adverse effects. The nonsignificant reduction in sleep latency suggests that LFWE may be less suited for acute sleep induction, but potentially valuable in sustaining sleep quality over prolonged use. This pharmacological profile parallels that of melatonin supplements, which primarily enhance circadian alignment rather than induce rapid sedation.^54^ However, unlike isolated melatonin, LFWE contains a complex mixture of phytochemicals that may exert synergistic effects on both neurochemical signaling and oxidative stress pathways. Given its water solubility, favorable safety profile and traditional use as a calming agent, LFWE holds promise for development into consumer health formats such as herbal infusions, nutraceutical capsules or functional beverages targeting mild to moderate sleep disturbances. Importantly, longan flowers are often discarded as agricultural waste during fruit production in many regions. Their conversion into functional sleep‐supportive ingredients may enhance resource utilization and create added value within longan production systems. These preclinical findings lay a scientific foundation for further translational research and support the potential of LFWE in functional sleep‐supportive formulations.

## FUNDING INFORMATION

This research was funded by grants NSTC 113‐2320‐B‐A49‐031‐MY3 from the Ministry of Science and Technology; 112NYCU‐TARI_01 from the Taiwan Agricultural Research Institute; and 2022SKHAND005 and 2025SKHAND012 from Shin Kong Wu Ho‐Su Memorial Hospital, Taipei, Taiwan.

## CONFLICT OF INTEREST

The authors declare no conflict of interest.

## AUTHOR CONTRIBUTIONS

Conceptualization, C‐JC, H‐FL and S‐TT; methodology and investigation, W‐CC and T‐LH; writing – original draft preparation, C‐PC and H‐KL; investigation, Y‐HL; writing – review and editing, project administration and funding acquisition, T‐HH and CH. All authors have read and agreed to the published version of the manuscript.

## ETHICS STATEMENT

All animal experiments, including mouse manipulations (e.g. dosing and bleeding), were performed under a protocol (IACUC permit number 1121005) approved by the Animal Research Committee of the National Yang Ming Chiao Tung University, Taipei, Taiwan. All plant materials were obtained through regulated commercial channels in accordance with Taiwan's Agricultural Market Transaction Act (Article 21, Paragraph 2). No special permits were required for this method of procurement.

## Data Availability

The datasets used and/or analyzed during the current study are available from the corresponding author on reasonable request.
